# CTX-M β-Lactamases in *Escherichia coli* from Community-acquired Urinary Tract Infections, Cambodia

**DOI:** 10.3201/eid1505.071299

**Published:** 2009-05

**Authors:** Etienne Ruppé, Sopheak Hem, Sovannarith Lath, Valérie Gautier, Frédéric Ariey, Jean-Louis Sarthou, Didier Monchy, Guillaume Arlet

**Affiliations:** Institut Pasteur du Cambodge, Phnom Penh, Cambodia (E. Ruppé, S. Hem, S. Lath, F. Ariey, J.-L. Sarthou, D. Monchy); Université Pierre et Marie Curie-Paris Faculté de Médecine, Paris, France (V. Gautier, G. Arlet); Assistance Publique-Hôpitaux de Paris–Hôpital Tenon, Paris (V. Gautier, G. Arlet)

**Keywords:** Antimicrobial resistance, bacteria, enteric infections, Escherichia coli, community, urinary tract infections, β-lactamase resistance, developing countries, Cambodia, research

## Abstract

Despite the recent global spread of CTX-M β-lactamases in *Escherichia coli* isolates from community-acquired urinary tract infections (CA-UTIs), their dissemination has been little studied in developing countries. In a 2-year prospective study, we documented the prevalence of extended-spectrum β-lactamases (ESBLs) in *E. coli* that were responsible for CA-UTIs in Phnom-Penh, Cambodia. Ninety-three *E. coli* strains were included. We observed a high prevalence of resistance to amoxicillin (88.2% of strains), cotrimoxazole (75.3%), ciprofloxacin (67.7%), gentamicin (42.5%), and third-generation cephalosporins (37.7%). A total of 34 strains carried ESBLs, all of which were CTX-M type. CTX-M carriage was associated with resistance to fluoroquinolones and aminoglycosides. U using repetitive extragenic palindromic–PCR, we identified 4 clusters containing 9, 8, 3, and 2 strains. The prevalence of CTX-M β-lactamases has reached a critical level in Cambodia, which highlights the need for study of their spread in developing countries.

## Abstract 2

The prevalence of CTX-M β-lactamases has reached a critical level, which highlights the need for study of their spread in developing countries.

*Escherichia coli* is the bacterium most frequently isolated in community- and hospital-acquired urinary tract infections (CA-UTIs and HA-UTIs, respectively). Despite possessing the gene encoding cephalosporinase, *ampC* (*1*), wild strains of *E. coli* are susceptible to most β-lactams because of the absence of an efficient *ampC* promoter region. The extensive use of β-lactam antimicrobial drugs has led to the emergence of resistant strains worldwide. β-lactam resistance is mostly mediated through acquisition of β-lactamase genes located on mobile genetic elements such as plasmids or transposons. Most β-lactamases found in *E. coli* belong to Ambler class A and can be further divided into narrow-spectrum β-lactamases (e.g., TEM-1, TEM-2, and SHV-1) and extended-spectrum β-lactamases (ESBLs) (e.g., TEM-3, SHV-5, and CTX-M-like) (*1*–*3*). ESBLs confer resistance to extended-spectrum cephalosporins, widely used to treat *E. coli* infections.

CTX-M–type β-lactamases (CTX-Ms) are broad-spectrum β-lactamases derived from the chromosomally encoded β-lactamases of *Kluyvera* sp. (*4*–*6*). So far, >70 CTX-M types have been isolated (www.lahey.org/studies, updated October 2008); these have been divided into 5 clusters on the basis of amino acid sequence: CTX-M-1, CTX-M-2, CTX-M-8, CTX-M-9, and CTX-M-25. Native CTX-Ms are cefotaximases that usually hydrolyze cefotaxime rather than ceftazidime. However, point mutations can extend their target spectrum to ceftazidime. Thus, CTX-M-15 and CTX-M-27 are derived by a single Asp240Gly substitution from CTX-M-3 and CTX-M-14, respectively (*7*,*8*).

Until recently, most ESBLs in clinical samples came from a hospital environment, belonged to the TEM or SHV β-lactamase family, and were produced by *Klebsiella* spp., *Enterobacter* spp., and *E. coli* (*9*). Within the past few years, the nature of ESBL dissemination has changed: *E. coli* is now the most frequently isolated ESBL-carrying bacterium, and CTX-Ms have become the most frequently isolated ESBLs (*10*). Moreover, in the last few years CTX-M–type ESBLs have emerged within the community, particularly among *E. coli* isolated from UTIs (*11*–*16*). Risk factors for CTX-M carriage in the community are largely unknown. CTX-Ms have been isolated from patients with CA-UTIs who have neither had recent antimicrobial drug treatment nor been admitted to a hospital or long-term care facility (*17*–*19*). Resistance to fluoroquinolones, aminoglycosides, and cotrimoxazole is often associated with CTX-M production (*16*,*17*), thereby limiting the choice of effective antimicrobial drugs to carbapenems or colistin.

Some studies have shown high prevalence of ESBLs among various *Enterobacteriaceae* in hospitals in developing countries (*20*–*22*). However, little information is available regarding prevalence within the community. Few bacteriologic data are available for Cambodia, and no studies of resistance of *Enterobacteriaceae* to antimicrobial agents have been reported. We investigated *E. coli* CA-UTIs in a 2-year prospective study. Our aims were 1) to establish the prevalence of community-acquired urinary *E. coli* resistance to a wide range of antimicrobial drugs and 2) to characterize the mechanisms underlying *E. coli* resistance to β-lactams.

## Methods

### Clinical Isolates from Patient Samples

Our laboratory at the Institut Pasteur in Phnom Penh, Cambodia, is a community laboratory, involved in various biological analyses. We receive biological samples from outpatients and hospitals that do not have bacteriology laboratories. Included in the study were patients who visited our laboratory with a suspected UTI from January 2004 through December 2005. Currently hospitalized patients were excluded, yet previous hospitalization was not an exclusionary criterion. We collected basic clinical data for each patient, including age and gender, whether antimicrobial drugs had been taken within the month preceding the sample submission, UTI history, and recent hospital visit. A UTI was defined by >10^4^ leukocytes/mL urine and >10^5^ CFU/mL urine. We used CLED (cystine-lactose-electrolyte deficient) agar (Dynamic Pharma, Phnom-Penh, Cambodia) for culture. Bacteria were identified with the API20E identification gallery (bioMérieux, Marcy l’Etoile, France). Strains were named (CEC [Cambodian *Escherichia coli*]) and numbered independently from isolation date.

### Antimicrobial Drug Susceptibility Testing and ESBL Confirmatory Testing

We determined antimicrobial drug susceptibility by the disk-diffusion method on Mueller-Hinton agar plates (Bio-Rad, Marnes-la-Coquette, France), as recommended by the French Society for Microbiology (www.sfm.asso.fr). We tested the following antimicrobial agents: amoxicillin, amoxicillin/clavulanic acid (coamoxiclav), ticarcillin, ticarcillin/clavulanic acid, piperacillin, piperacillin/tazobactam, cefalothin, cefoxitin, cefotaxime, ceftazidime, cefepime, imipenem, moxalactam, aztreonam, nalidixic acid, norfloxacin, ciprofloxacin, gentamicin, tobramycin, netilmicin, amikacin, nitroxolin, fosfomycin, and sulfamethoxazole/trimethoprim (cotrimoxazole). We detected ESBLs by using the double-disk synergy test (clavulanic acid and cefotaxime, ceftazidime, cefepime and aztreonam) performed on Mueller-Hinton media. We determined MICs for cefoxitin, cefotaxime, ceftazidime, and cefepime by using the agar-dilution method for ESBL-carrying strains and strains with decreased susceptibility to cefoxitin (as interpreted by the disk-diffusion method) as recommended by the French Society for Microbiology. For cefoxitin, strains were considered susceptible if MICs were <8 mg/L; intermediate susceptible if MICs were 16–32 mg/L, and resistant if MICs were >32 mg/L. For cefotaxime, strains were considered susceptible if MICs were <1 mg/L; intermediate susceptible if MICs were 2 mg/L, and resistant if MICs were >2 mg/L. For ceftazidime and cefepime, strains were considered susceptible if MICs were <4 mg/L, intermediate susceptible if MICs were 8 mg/L, and resistant if MICs were >8 mg/L.

### PCR Amplifications

Template DNA was prepared by boiling. Briefly, 5 colonies were suspended thoroughly in 1 mL DNase- and RNase-free water and boiled for 10 min. After centrifugation, supernatant was used as template DNA. We amplified the *ampC* upstream region, *bla*_TEM_, *bla*_SHV_, *bla*_CTX-M_, *bla*_VEB,_ *bla*_OXA-1_, and *bla*_CMY_ by PCR, using specific oligodeoxynucleotides (Table 1). PCR was performed in a 25-µL mixture of 1× buffer (supplied with *Taq* polymerase), 2.5 mmol/L MgCl_2_, 2.5 U of FIREPol DNA polymerase (Solis BioDyne, Tartu, Estonia), 200 µmol/L of each deoxynucleoside triphosphate, and 25 pmol of each primer. The PCR mixture was subjected to a 5-min denaturation step at 94°C, followed by 30 cycles of 45 s at 94°C, 45 s at 55°C, and 60 s at 72°C, and a final elongation step of 5 min at 72°C. PCR products were separated by 100-V electrophoresis in a 2% agarose gel for 30 min, after which they were stained with ethidium bromide.

**Table 1:** Primers used to study CTX-M β-lactamases in *Escherichia coli,* Cambodia, 2004–2005

Gene detected	Primer name	Primer sequence (5′ → 3′)	Reference
*bla_TEM_*	C	TCG GGG AAA TGT GCG CG	(*23*)
	D	TGC TTA ATC AGT GAG GCA CC	
*bla_SHV_*	OS-5	TTA TCT CCC TGT TAG CCA CC	(*24*)
	OS-6	GAT TTG CTG ATT TCG CTC GG	
*bla_CTX-M_*	MA-1	SCS ATG TGC AGY ACC AGT AA	(*23*)
	MA-2	CCG CRA TAT GRT TGG TGG TG	
*bla_CTX-M_* group 9	M9U	ATG GTG ACA AAG AGA GTG CA	(*23*)
	M9L	CCC TTC GGC GAT GAT TCT C	
*bla_CTX-M_* group 1	M13U	GGT TAA AAA ATC ACT GCG TC	(*23*)
	M13L	TTG GTG ACG ATT TTA GCC GC	
*bla_ampC_*	AmpC1	AAT GGG TTT TCT ACG GTC TG	(*25*)
	AmpC2	GGG CAG CAA ATG TGG AGC AA	
*bla_VEB_*	casF	CGA CTT CCA TTT CCC GAT GC	(*26*)
	casB	GGA CTC TGC AAC AAA TAC GC	
*bla_OXA-1_*	OXA-1up	TAT CAA CTT CGC TAT TTT TTT A	(*27*)
	OXA-1low	TTT AGT GTG TTT AGA ATG GTG A	
*bla_CMY_*	CF1	ATGATGAAAAAATCGTTATGC	(*28*)
	CF2	TTGTAGCTTTTCAAGAATGCGC	
*chuA*	ChuA.1	GAC GAA CCA ACG GTC AGG AT	(*29*)
	ChuA.2	TGC CGC CAG TAC CAA AGA CA	
*ygaA*	YgaA.1	TGA AGT GTC AGG AGA CGC TG	(*29*)
	YgaA.2	ATG GAG AAT GGG TTC CTC AAC	
*TspE4C2*	TspE4C2.1	GAG TAA TGT CGG GGC ATT CA	(*29*)
	TspE4C2.2	CGC GCC AAC AAA GTA TTA GC	

### PCR Product Sequencing

Amplification products were purified with Montage PCR Filter Units (Millipore, Billerica, MA, USA). Sequencing reactions were performed in a PTC-225 Peltier Thermal Cycler (MJ Research, Waltham, MA, USA) by using an ABI PRISM BigDye Terminator Cycle Sequencing Kit with AmpliTaq DNA polymerase (Applied Biosystems, Branchburg, NJ, USA), according to the manufacturer’s instructions. Each template was sequenced with the appropriate primer. Fluorescence-labeled fragments were purified from the unincorporated terminators with an ethanol precipitation protocol. The samples were resuspended in distilled water and subjected to electrophoresis in an ABI 3730xl sequencer (Applied Biosystems).

### Phylogenetic Group Determination

To determine phylogenetic group (i.e., A, B1, B2, and D), we performed triplex PCR for all strains (n = 93) as described previously (*29*). We used *chuA* and *yjaA* genes and an *E. coli* DNA fragment, TSPE4.C2.

### Repetitive Extragenic Palindromic PCR

The clonality of ESBL-positive strains was assessed by repetitive extragenic palindromic (rep)–PCR. DNA was extracted by using the QIAGEN Mini kit (QIAGEN, Courtaboeuf, France). REP-PCRs were performed for all strains in the same batch with primers rep-1R and rep-2T, as described previously (*28*). The resulting products were separated by 70-V electrophoresis in a 1% agarose gel for 3 h, after which they were stained with ethidium bromide. Strains with suspected similar migration profiles were then migrated together with the ESBL-positive strains to assess their similarity. Photographs of the gels (Figure) have been harmonized to be more informative.

**Figure:**
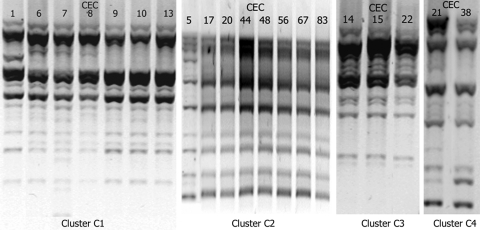
Results of repetitive extragenic palindromic–PCR of *Escherichia coli* isolates belonging to the 4 clusters, Cambodia, 2004–2005. CEC, Cambodian *E*. *coli*.

### Statistical Analysis

Data were analyzed by using Epi Info version 3.2 (www.cdc.gov/epiinfo). Risk factors for ESBL-producing *E. coli* were assessed by using univariate analysis with the χ^2^ or Fisher exact tests; however, we used analysis of variance to determine whether age had an association with ESBL-producing *E. coli*. Associations between ESBL type, co-resistance, mutations in *ampC* promoter regions, and phylogenetic groups were separately tested by using the χ^2^ or Fisher exact tests. The significance threshold was 0.05.

## Results

### Population Characteristics

Of the 861 urine samples, 194 were positive for UTI and 163 contained *E. coli.* Samples from hospitalized patients and samples isolated from the same patient in a short period were excluded from the study. Overall, 93 patients with *E. coli*–related CA-UTIs were recruited for the study, all of whom were living in Phnom Penh. Their mean age was 43 years (range 1–88 years, median 40 years), and the M:F ratio was 0.21. No pregnant women were included. Twenty-eight (30.1%) patients had a history of UTI (with no details available), 24 (25.8%) had taken antimicrobial drugs, and 9 (9.7%) had sought consultation at a hospital in the month preceding the urine sampling. Patients with a history of UTI had taken significantly more antimicrobial drugs than had persons without UTIs (p<0.0001).

### Prevalence of Antimicrobial Drug Resistance in *E. coli* Strains

Disk-diffusion susceptibility testing indicated high prevalence of resistance to various antimicrobial agents: 88 (94.6%) strains were resistant to amoxicillin and ticarcillin, and 20 (21.5%) were immediate susceptible to cefoxitin. We observed synergy between clavulanic acid, oxyimino-cephalosporins, and aztreonam for 34 strains (36.6%). Decreased susceptibility to cefoxitin was significantly associated with ESBL expression (p<0.001). All strains (n = 93) were susceptible to imipenem. One strain (CEC93) showed a high level of resistance to cefoxitin, cefotaxime, and ceftazidime but not to cefepime; double-disk synergy test results were negative for clavulanate. We observed a substantial level of resistance to quinolones; 63 strains (67.7%) had an intermediate level of resistance or were fully resistant to nalidixic acid, norfloxacin, and ciprofloxacin. Quinolone resistance was strongly associated with ESBL expression (p<0.0001). Of the 34 ESBL-carrying strains, only 2 were susceptible to quinolones. Cotrimoxazole resistance was observed in 70 strains (75.3%) but was not significantly associated with ESBL expression (p = 0.06). Aminoglycoside resistance was also significantly associated with ESBL expression (p<0.01). Thirty-nine strains (41.9%) were resistant to at least 1 of the 4 tested aminoglycosides. The most frequently observed phenotypic profile included resistance to gentamicin, tobramycin, and netilmicin (37.6% of the strains). None of the strains were resistant to fosfomycin or nitrofurantoin (Table 2).

**Table 2:** Resistance to antimicrobial agents among ESBL-positive and ESBL-negative *Escherichia coli,* Cambodia, 2004–2005*

Antimicrobial agent	Resistance ratios, % (no. resistant strains)			p value
	ESBL+, n = 34	ESBL–, n = 59	Total, n = 93	
Fluoroquinolones	94 (32)	53 (31)	68 (63)	<0.001
Cotrimoxazole	85 (29)	69 (41)	75 (70)	0.06
Aminoglycosides	65 (22)	27 (16)	42 (39)	<0.01
Co-amoxiclav	94 (32)	19 (11)	46 (43)	<0.001
Cefoxitin	41 (13)	11 (7)	22 (20)	<0.001
Nitrofurantoin	0	0	0	NS
Fosfomycin	0	0	0	NS

*ESBL, extended-spectrum β-lactamase; NS, not significant.

### Risk Factors for ESBL Carriage

Univariate analysis showed no risk factors for ESBL carriage. Carriage was not significantly associated with gender, age, previous hospitalization (within the past month), antimicrobial drug treatment (within the last month), or history of UTI.

### β-Lactamase Characterization

Thirty-four strains were tested for ESBL identification. They were all positive for *bla*_CTX-M_ and negative for *bla*_VEB_ and *bla*_SHV_. We detected *bla*_TEM_ in 26 (76.4%) of the ESBL-carrying strains. CTX-M-14 was the most frequently isolated ESBL (n = 15), followed by CTX-M-27 (n = 12) and CTX-M-15 (n = 5). One strain (CEC7) was carrying both *bla*_CTX-M-14_ and *bla*_CTX-M-15_. Strain CEC14 was carrying a *bla*_CTX-M-14_ variant, which differed from the parental enzyme by a single transversion (C825G) leading to a Ser273Arg amino acid change. *bla*_TEM-1_ was detected in 24 (96%) of the *bla*_TEM_-carrying strains. One strain (CEC23) had a DNA sequence that differed from TEM-1 by 4 nucleotides. Only 1 of these mutations resulted in an amino acid change (Met182Thr). Amino acid sequence comparison (www.lahey.org/studies/webt.asp) showed that this TEM had the same amino acid sequence as TEM-135.

Strain CEC93, exhibiting a phenotypic profile indicative of a high level of cephalosporinase expression, was positive for *bla*_CMY_. Sequencing confirmed the presence of *bla*_CMY-2_ in this strain. We detected *bla*_OXA-1_ in 4 CTX-M-15–producing strains: CEC15, CEC48, CEC68, and CEC89.

### *ampC* Sequencing Results

Strains with different levels of resistance to cefoxitin, ranging from intermediate (cefoxitin MIC = 8 mg/L) to full resistance (cefoxitin MIC >8 mg/L), had different mutations within the region upstream from *ampC* (Table 3). Only 1 strain did not display any mutations. Most mutations occurred within the transcriptional attenuator region; the most frequently observed profiles were +22 C>T, +26 T>G, +27 A>T, +32 G>A, and +70 C>T (11 strains). The other strains each exhibited a different profile. Strain CEC57 had the profile –32 T>A with an additional nucleotide change, which significantly increases *ampC* expression (*31*). Strain CEC92 had the combination –42 C>T and –28 G>A, which also increases *ampC* expression (*25*).

**Table 3:** Mutations detected in the *ampC* promoter region of CTX-M β-lactam–resistant Escherichia coli*

Isolate	CEF MIC, mg/L	CTX-M	Phylogenetic group	Mutation at position†											
				−42	−32	−28	−18	−1	+22	+26	+27	+32	+58	+70	+81
*E. coli* K12 (U00096)	–	–	–	C	T	G	G	C	C	T	A	G	C	C	G
CEC1	16	Yes	D						T	G	T	A		T	
CEC3	32	No	D						T	G	T	A		T	
CEC4	32	No	D				A	T					T		
CEC6	32	Yes	D						T	G	T	A		T	
CEC8	32	Yes	D						T	G	T	A		T	
CEC9	32	Yes	D						T	G	T	A		T	
CEC10	32	Yes	D						T	G	T	A		T	
CEC13	32	Yes	D						T	G	T	A		T	
CEC14	16	Yes	D											T	
CEC15	16	Yes	D											T	
CEC19	32	No	D						T	G	T	A		T	
CEC21	16	Yes	D			A							T		A
CEC22	16	Yes	D											T	
CEC31	16	Yes	D			A							T		A
CEC57	32	No	D		A				T	G	T	A		T	
CEC88	32	No	B2			A									A
CEC89	32	Yes	D						T	G	T	A		T	
CEC92	>128	Yes	D	T			A	T					T		
CEC93	>128	No	D						T	G	T	A		T	

*CEF, cefoxitin; CEC, Cambodian *E*. *coli*. †According to numbering of Jaurin and Grundström (*30*).

### Phylogenetic Groups

Most of the strains studied belonged to groups B2 (53%; n = 49) and D (28%; n = 26), as do most pathogenic *E. coli* (*32*). Twelve strains belonged to group A (13%) and 6 to group B1 (6%). Among ESBL-carrying strains, 53% belonged to group B2 (n = 18), 38% to D (n = 13), 6% to A (n = 2), and 3% to B1 (n = 1). Resistance to cotrimoxazole was less prevalent in B2 strains than in strains from the other groups (p<0.05). Resistance to quinolones was more prevalent among group D strains than among strains from other groups (p<0.005). Furthermore, a strong association (p<0.0001) was found between decreased susceptibility to cefoxitin and group D strains. No association was shown between phylogenetic group and other characteristics such as ESBL carriage and aminoglycoside resistance. Among ESBL-carrying strains, none of the 4 groups were associated with 1 particular type of CTX-M.

### Rep-PCR Findings

Twenty-one ESBL positive strains and the CMY-2–producing strain were divided into 4 clusters; the remaining strains were genetically unrelated (Appendix Table; Figure). Cluster C1 consisted of 8 strains with an identical *ampC* mutation profile, all belonging to phylogenetic group D. However, β-lactamase content was variable: 6 strains harbored CTX-M-27 and TEM-1, 1 strain harbored only CMY-2, and 1 harbored only CTX-M-15. Strain CEC7 (belonging to group B2, carrying CTX-M-14 and CTX-M-15, with no *ampC* mutations) exhibited a similar REP-PCR profile to that of strains in cluster C1. Cluster C2 consisted of 8 strains belonging to group B2 with no *ampC* mutations and carrying various β-lactamases; 6 strains produced CTX-M-14 (2 of which co-produced TEM-1), 1 produced CTX-M-27/TEM-1, and 1 produced CTX-M-15/TEM-1/OXA-1. Cluster C3 consisted of 3 strains from group D, all with the same *ampC* mutation profile, but with 3 distinct β-lactamase profiles (CTX-M-14 and TEM-1; CTX-M-14-like and TEM-1; and CTX-M-15 and TEM-1). Cluster C4 consisted of 2 strains from group D, each with the same *ampC* mutation profile but different β-lactamase profiles (CTX-M-14 and TEM-1; and CTX-M-27 only).

## Discussion

We surveyed antimicrobial drug resistance in Cambodia. Our prospective study in this developing country focused on CTX-Ms CA-UTIs caused by *E. coli*. Although the patients included came from the community, antimicrobial drug resistance was prevalent among UTI-causing strains, particularly to β-lactams (including extended-spectrum cephalosporins). Our findings suggest that CTX-M-type β-lactamases are widespread in Cambodia. CTX-M production was significantly associated with resistance to quinolones and aminoglycosides and with decreased susceptibility to cefoxitin, leading to a high prevalence of multiresistant strains. The spread of CTX-M in the community has already been described through prospective studies in industrialized countries such as Canada (*33*), France (*34*), and the United Kingdom (*12*); however, we found higher prevalence of CTX-Ms in Cambodia than that reported in these previous studies.

We propose 3 possible explanations for the situation in Cambodia. First, although no reliable data were available, it is well known that many persons in the community self-medicate or obtain prescriptions for non-adapted drugs (drugs taken without any antibiotic susceptibility testing of the strain that causes the infections); these practices suggest that uncontrolled consumption of antimicrobial agents is likely to play a major role. This factor, together with the likely substandard quality of some drugs, undoubtedly contributes to the high prevalence of resistance. Second, hygiene in Cambodia is poor, and the population density in Phnom Penh is high. The saturated sewer system, particularly during the rainy season, likely facilitates efficient propagation and spread of bacteria within the community. Grenet et al. proposed that that poor hygiene in a French-Guyanese Indian community led to the spread of resistant bacteria despite low antibiotic pressure (*35*). Similarly, poor hygiene conditions may have led to the spread of resistant strains in the Cambodian community studied here. The TEM-type and SHV-type ESBL—until recently the predominant ESBL family subtypes—have never been implicated in the spread of ESBL in the community. Why CTX-M strains are the only ESBL types to spread in the community is not clear. CTX-M strains may have a particularly high capacity to disseminate or an ecologic advantage over other ESBLs and thus persist in the community, whereas other ESBL may be progressively eliminated with decreasing antibiotic pressure.

One limitation to our study is that patients at our institute are not necessarily representative of the Cambodian population. For most persons in Cambodia, antimicrobial agents and biologic analyses are expensive. Therefore, our patients may have been wealthier and may have taken more courses of antimicrobial drug treatment than other Cambodians.

REP-PCR yielded 4 clusters of strains, consistent with their identical *ampC* mutational profiles, yet with various contents of β-lactamases. Because all included patients were living in Phnom Penh, mediocre hygiene might have favored the diffusion of clones within an urban area, a phenomenon that had previously been observed in large-scale studies undertaken in the United Kingdom (*12*), Canada (*36*), Italy (*37*), and Brazil (*15*) but not in Hong Kong (*13*). In contrast to our results, clones identified in those studies harbored the same β-lactamases. Further investigation using multilocus sequence typing would be necessary to identify the molecular determinants of the CTX-M–carrying *E. coli* pandemic in Cambodia.

Branger et al. have observed that group D *E. coli* were more frequently resistant to quinolones (*38*) than were non-D *E. coli*; these findings are consistent with our results. Moreover, we found a strong association (p<0.001) between group D strains and decreased susceptibility to cefoxitin (secondary to the effect of mutations in the *ampC* promoter region). This association was also present when all strains were taken into account (p<0.001). However, although CTX-M carriage was more frequently observed for group D strains than for non-D strains, the association was not significant. Given that quinolone resistance and *ampC* hyperexpression involve several mutations, a possible explanation for this association with group D strains may be a stronger mutation capacity for these strains than for strains belonging to other groups. Further investigations will be required to explain this phenomenon.

Community-isolated ESBL-carrying strains are an emerging challenge for community practitioners and hospitals. Information is not readily available in either developing countries or in industrialized countries, and UTI treatment guidelines remain unchanged. In Cambodia, and probably in many other developing countries, resistant *E. coli* strains are endemic to the community. Investigating the current situation in Cambodia may improve our understanding of the situation in industrialized countries, where ESBLs are no longer uncommon in the community. According to our experience in Cambodia, measures should focus on improving hygiene and appropriate prescribing of antimicrobial agents. In conclusion, we suggest that this high prevalence of β-lactam resistance in Cambodia is due to the intrinsic capacity of CTX-M–encoding genes to disseminate through communities where hygiene and living conditions are poor and antimicrobial drug consumption is uncontrolled.

## Supplementary Material

Appendix Table Characterization of the ESBL- or plasmidic ampC-type _-lactamase-carrying Escherichia coli strains (N = 35), Cambodia, 2004-2005*
