# Supplementary material for: CTX-M β-Lactamases in Escherichia coli from Community-acquired Urinary Tract Infections, Cambodia
Source: Emerg Infect Dis. 2009 May;15(5):741–8. doi: 10.3201/eid1505.071299 (PMC2687024; doi:10.3201/eid1505.071299)
Supplement: Appendix Table — Characterization of the ESBL- or plasmidic ampC-type _-lactamase-carrying Escherichia coli strains (N = 35), Cambodia, 2004-2005* [file 07-1299_appT-s1.pdf]

Appendix Table. Characterization of the ESBL- or plasmidic *ampC*–type β-lactamase–carrying *Escherichia coli* strains (N = 35), Cambodia, 2004–2005*

E. coli strain no.	Phylogenetic group	*ampC* mutations†	CTX-M type	TEM type	OXA type	CMY type	MICs (µg/mL)				Other antimicrobial agents to which resistance was found‡	Rep-PCR cluster
							CAZ	CTX	FEP	FOX		
CEC1	D	+22 +26 +27 +32 +70	27	TEM-1	–	–	16	128	16	16	GEN, NAL, CIP, SXT	C1
CEC6	D	+22 +26 +27 +32 +70	27	TEM-1	–	–	32	>128	32	32	GEN, NAL, CIP, SXT	C1
CEC8	D	+22 +26 +27 +32 +70	27	TEM-1	–	–	32	>128	32	32	NAL, CIP	C1
CEC9	D	+22 +26 +27 +32 +70	27	TEM-1	–	–	16	>128	32	32	NAL, CIP	C1
CEC10	D	+22 +26 +27 +32 +70	27	TEM-1	–	–	8	128	8	32	GEN, NAL, CIP, SXT	C1
CEC13	D	+22 +26 +27 +32 +70	27	TEM-1	–	–	16	>128	32	32	NAL, CIP	C1
CEC89	D	+22 +26 +27 +32 +70	15	–	OXA-1	–	64	>128	64	32	GEN, AMK, NAL, CIP, SXT	C1
CEC93	D	+22 +26 +27 +32 +70	–	–	–	CMY-2	128	128	4	>128	GEN, NAL, CIP, SXT	C1
CEC5	B2	–	14	TEM-1	–	–	16	128	16	8	GEN, NAL, CIP, SXT	C2
CEC17	B2	–	14	TEM-1	–	–	2	128	16	4	GEN, NAL, CIP, SXT	C2
CEC20	B2	–	14	–	–	–	2	64	16	4	GEN, NAL, CIP, SXT	C2
CEC44	B2	–	14	–	–	–	2	128	32	8	NAL, CIP, SXT	C2
CEC48	B2	–	15	TEM-1	OXA-1	–	32	>128	32	4	GEN, NAL, CIP	C2
CEC56	B2	–	14	–	–	–	8	128	64	8	NAL, CIP, SXT	C2
CEC67	B2	–	27	TEM-1	–	–	8	128	8	8	NAL, SXT	C2
CEC83	B2	–	14	-	–	–	1	32	4	4	GEN, NAL, CIP, SXT	C2
CEC14	D	+70	14	TEM-1	–	–	2	128	4	32	NAL, CIP, SXT	C3
CEC15	D	+70	15	TEM-1	OXA-1	–	32	>128	32	16	GEN, NAL, CIP, SXT	C3
CEC22	D	+70	14	TEM-1	–	–	8	>128	64	16	GEN, NAL, CIP, SXT	C3
CEC21	D	−28, +58, +81	14	TEM-1	–	–	1	64	8	16	NAL, CIP, SXT	C4
CEC38	D	−28, +58, +81	27	–	–	–	8	128	8	16	NAL, CIP, SXT	C4
CEC18	B2	–	14	TEM-1	–	–	2	128	16	4	GEN, NAL, CIP, SXT	–
CEC2	B2	–	15	TEM-1	–	–	16	>128	32	2	SXT	–
CEC31	D	–	14	TEM-1	–	–	2	128	8	8	GEN, NAL, CIP, SXT	–
CEC39	B2	–	27	TEM-1	–	–	8	64	4	8	GEN, NAL, CIP, SXT	–
CEC40	B2	–	14	TEM-1	–	–	2	128	16	8	GEN, NAL, CIP, SXT	–
CEC41	B2	–	27	TEM-1	–	–	4	128	8	8	GEN, NAL, CIP, SXT	–
CEC_{50}	A	–	14	–	–	–	1	16	2	4	GEN, NAL, CIP, SXT	–
CEC55	A	–	14	TEM-1	–	–	2	64	8	8	GEN, NAL, CIP, SXT	–
CEC66	B2	–	14	TEM-1	–	–	8	128	32	8	GEN, NAL, CIP, SXT	–
CEC68	B2	–	15	TEM-1	OXA-1	–	32	128	16	8	GEN, NAL, CIP, SXT	–
CEC7	B2	–	14,15	–	–	–	32	>128	32	8	GEN, NAL, CIP, SXT	–
CEC75	B2	–	14	TEM-1	–	–	1	32	4	4	GEN, SXT	–
CEC90	B2	–	27	–	–	–	128	128	4	8	NAL, CIP	–
CEC92	B1	−42, −18, −1	27	TEM-1	–	–	128	>128	>128	>128	GEN, NAL, CIP, SXT	–

*ESBL, extended-spectrumβ-lactamase; Rep-PCR, repetitive extragenic palindromic PCR; CAZ, ceftazidime; CTX, cefotaxime; FEP, cefepime; FOX, cefoxitin; GEN, gentamicin; NAL, nalidixic acid; CIP, ciprofloxacin, SXT, cotrimoxazole; AMK, amikacin
†According to the numbering of Jaurin and Grundström (*30*).
